# Supplementary material for: Structure of full-length cobalamin-dependent methionine synthase and cofactor loading captured in crystallo
Source: Nat Commun. 2023 Oct 11;14:6365. doi: 10.1038/s41467-023-42037-4 (PMC10567725; doi:10.1038/s41467-023-42037-4)
Supplement: Supplementary file 1 — Supplementary Information [file 41467_2023_42037_MOESM1_ESM.pdf]

Structure of full-length cobalamin-dependent methionine synthase and cofactor loading captured *in crystallo*

Johnny Mendoza[1‡], Meredith Purchal[2,3‡], Kazuhiro Yamada[1,4*], Markos Koutmos[1,2,5*]

[1] Department of Chemistry, University of Michigan, Ann Arbor, MI, 48109

[2] Program in Chemical Biology, University of Michigan, Ann Arbor, MI, 48109

[3] New England Biolabs, Inc., Ipswich, MA, 01938

[4] Department of Biological Chemistry, University of Michigan, Ann Arbor, MI, 48109

[5] Program in Biophysics, University of Michigan, Ann Arbor, MI, 48109

‡ Johnny Mendoza and Meredith Purchal contributed equally to this work

*To whom correspondence should be addressed: yamadak@umich.edu (co-corresponding), mkoutmos@umich.edu (corresponding)

Supplementary Information

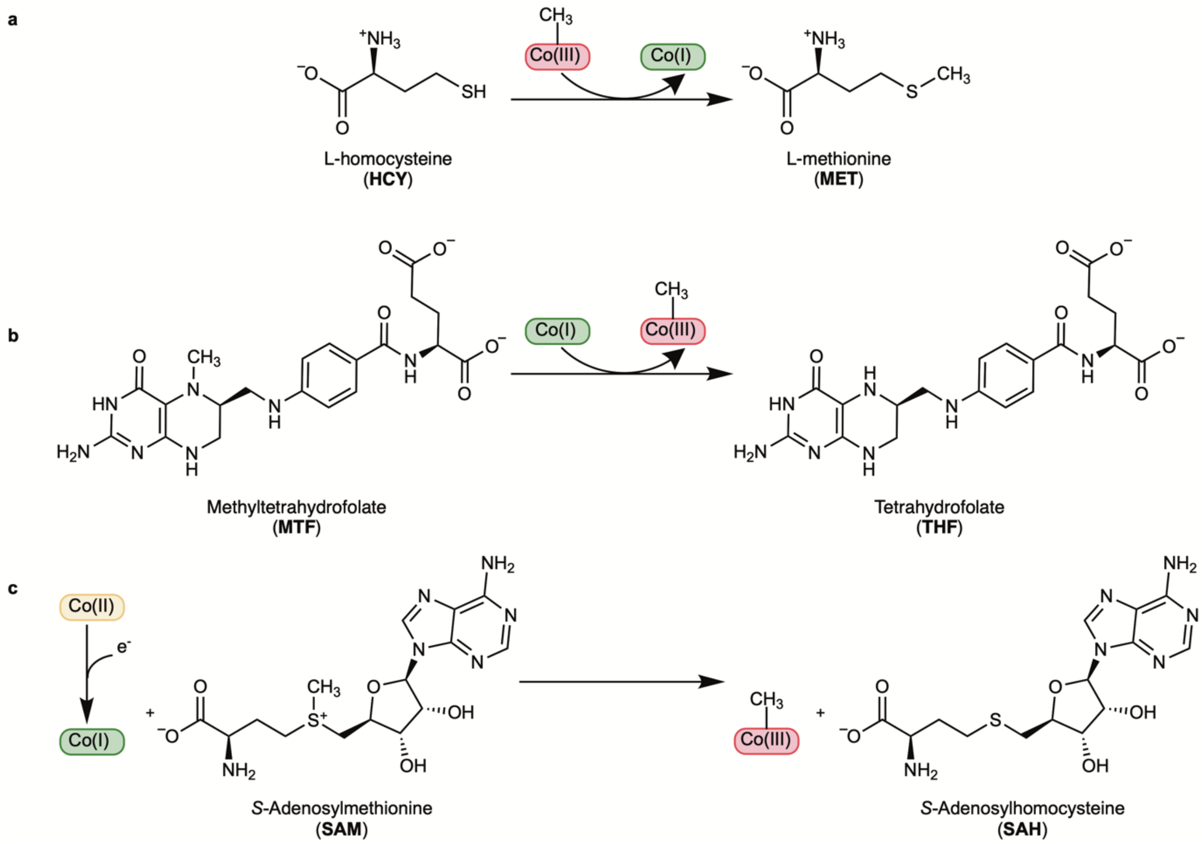

Supplementary Figure 1. The three methylations catalyzed by methionine synthase. **a** The methylation of homocysteine by MS using CH_3-Co(III) (MeCbl) to form methionine. **b** The demethylation of methyltetrahydrofolate by MS using Co(I) to yield CH_3-Co(III) (MeCbl). **c** The reactivation reaction catalyzed by MS, a reductive methylation and reactivation of Co(II) to yield CH_3-Co(III) (MeCbl).

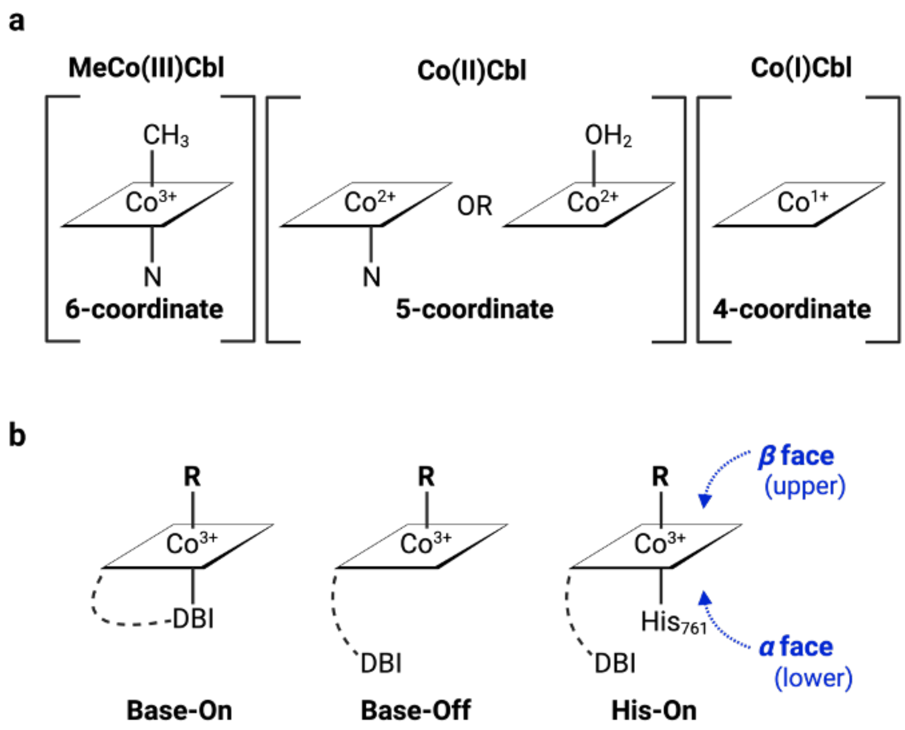

Supplementary Figure 2. Cobalamin coordination environment. a Coordination environment of cobalamin in different cobalt oxidation states. **b** Base-on/Base-off. In solution, the DBI tail is coordinated in the lower axial position. Upon binding, the DBI tail is replaced with His to achieve the His-on state.

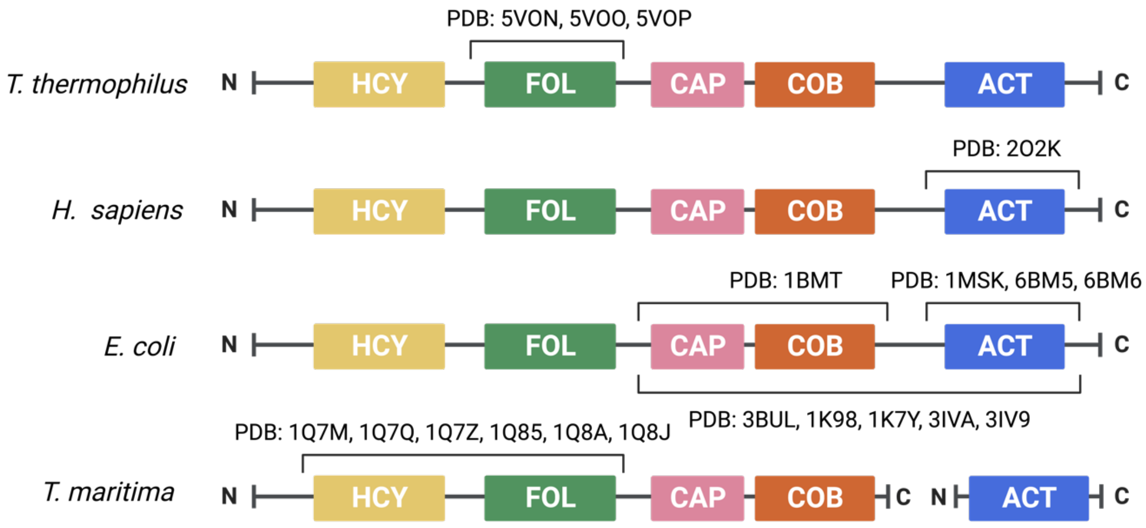

Supplementary Figure 3. MS domain organization and PDB structures. Domain organization of *T. thermophilus* MS and several MS homologs from *H. sapiens*, *E. coli*, and *T. maritima*. Structures corresponding to excised domains from each homolog are shown: *t*MS (5VON, 5VOO, 5VOP), *h*MS (2O2K), *e*MS (1BMT, 1MSK, 6BM5, 6BM6, 3BUL, 1K98, 1K7Y, 3IVA, 3IV9), and *T. martima* MS (1Q7M, 1Q7Q, 1Q7Z, 1Q85, 1Q8A, 1Q8J).

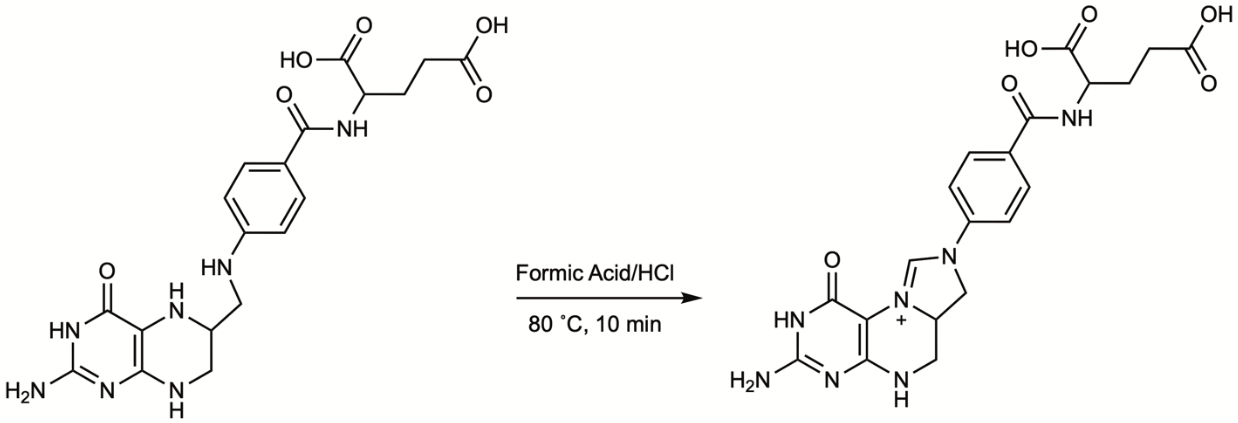

Tetrahydrofolate
(**THF**)

5,10-Methenyltetrahydrofolate
(**CH^+=H_4Folate**)
350 nm

Supplementary Figure 4. Acid-catalyzed conversion Tetrahydrofolate to methenyltetrahydrofolate. The resulting product has a characteristic absorbance at 350 nm and is used to track *t*MS activity in a coupled-assay described in Methods (Drummond, J. T., Jarrett, J., Gonzalez, J. C., Huang, S. & Matthews, R. G. Characterization of Nonradioactive Assays for Cobalamin-Dependent and Cobalamin-Independent Methionine Synthase Enzymes. *Anal. Biochem.* **228**, 323–329 (1995)).

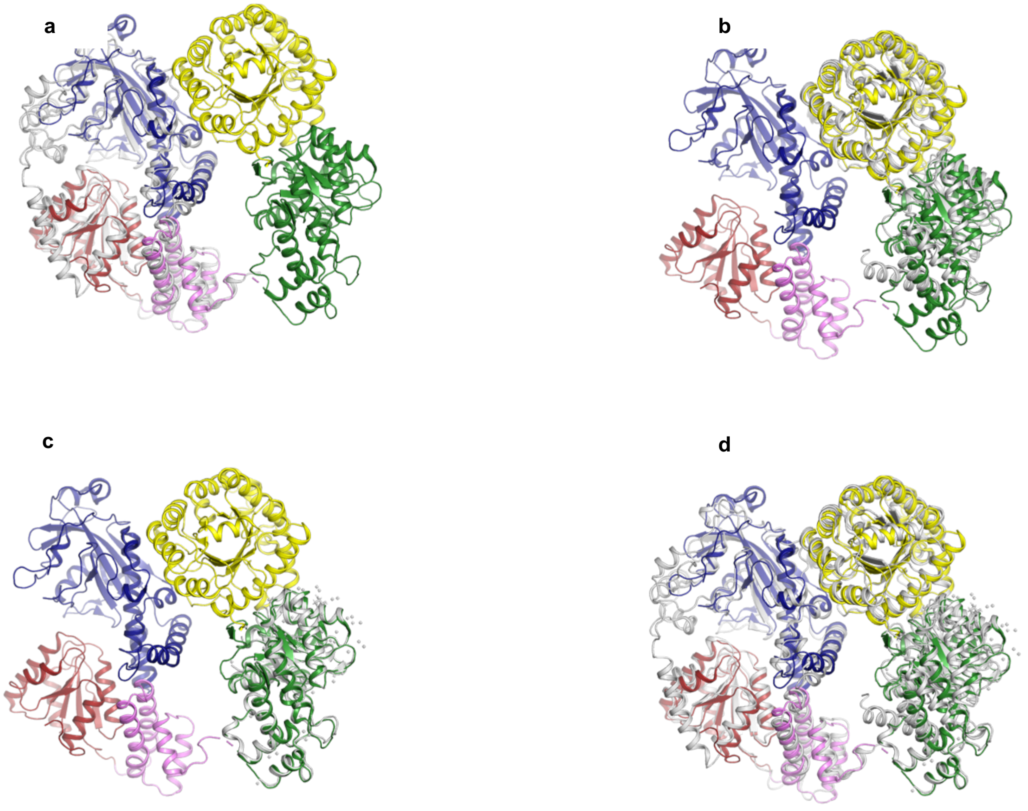

Supplementary Figure 5. Structural alignment of excised MS domains with full-length *t*MS. **a** Alignment of Cap:Cob:Act domains from 1K7Y (gray) with Cap (pink), Cob (red), and Act (blue) in full-length *t*MS – RMSD=2.16 Å. **b** Alignment of Fol and Hcy domains from 3BOL (gray) with the Fol (green) and Hcy (yellow) domains in full-length *t*MS – RMSD=2.64 Å. **c** Alignment of Fol 5VON (gray) domain with Fol domain (green) in full-length *t*MS – RMSD=0.46 Å. **d** Alignment of the excised domains (gray) from 1K7Y, 3BOL, and 5VON with full-length *t*MS.

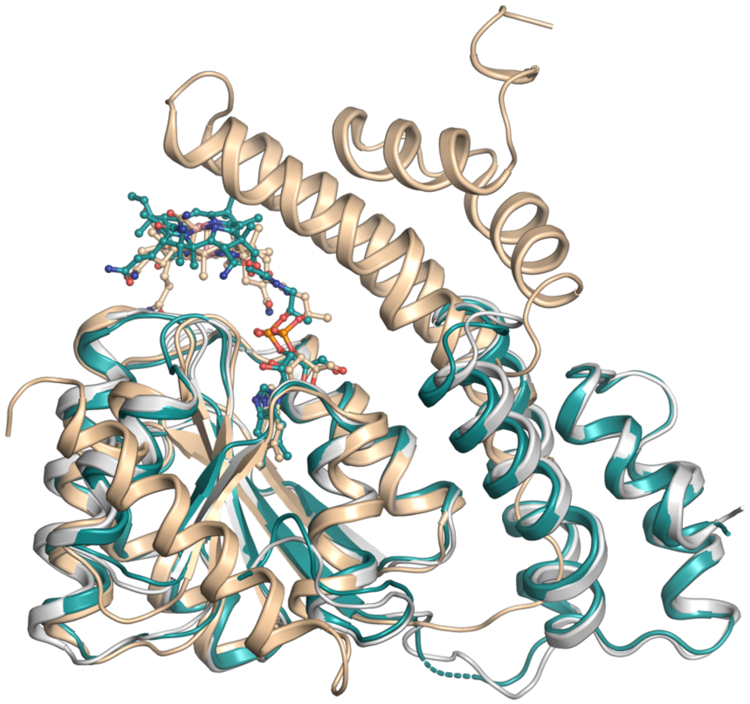

Supplementary Figure 6. **Displacement of Cap-domain in full length MS.** Alignment of Cap:Cob domains from the full length structure (gray, reactivation) with the Cob domain from *holo*-Cap:Cob:Act (teal, reactivation, His-off) – RMSD=0.72 Å and the Cap:Cob domains from 1BMT (wheat, reactivation, His-on) – RMSD=0.85 Å.

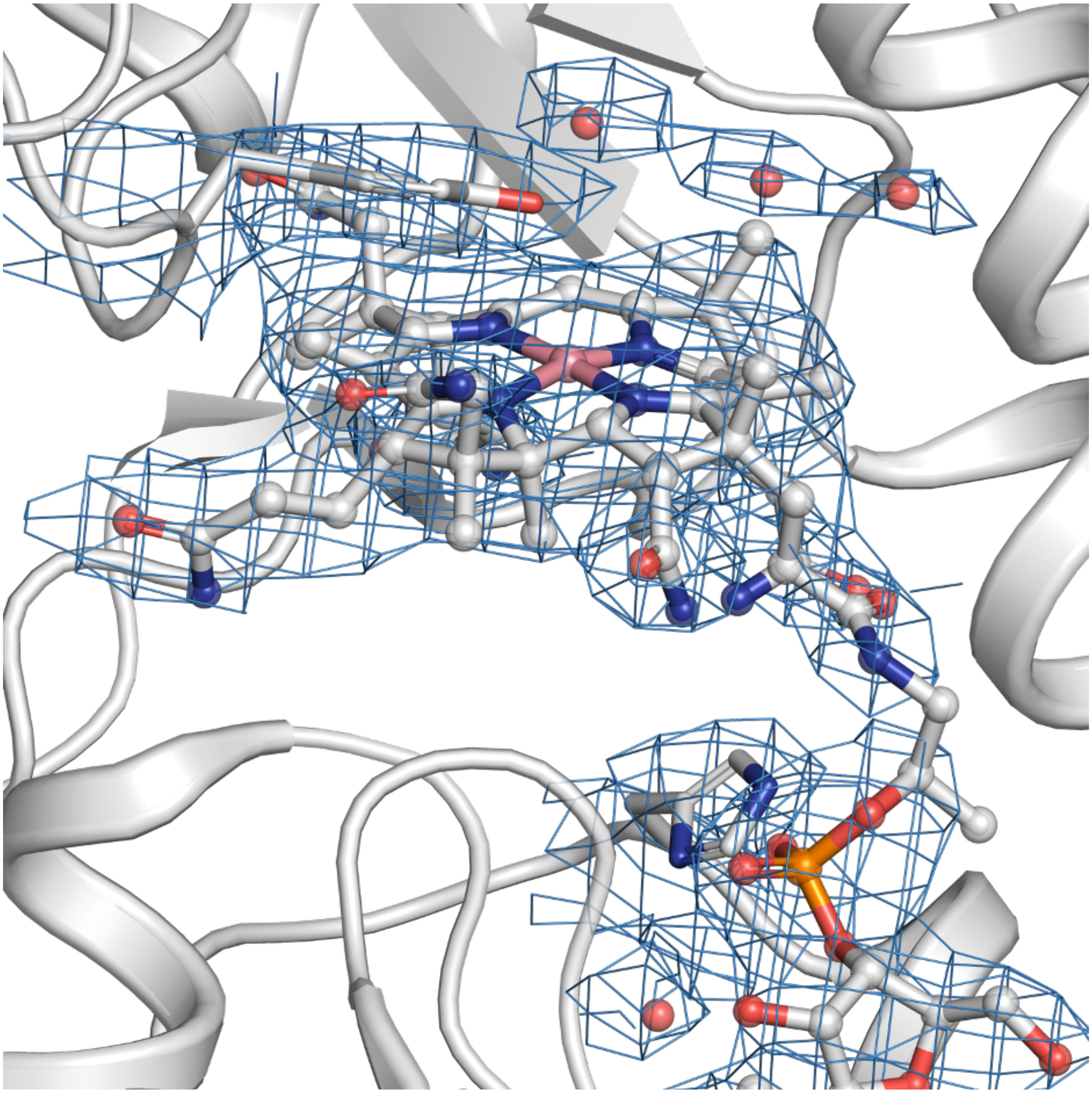

Supplementary Figure 7. *t*MS electron density around the cobalamin cofactor. *Holo*-Cap:Cob:Act (light pink) and its cobalamin cofactor (gray), along with Tyr1132 and His761 in the axial cofactor positions. Their corresponding electron density ($2F_o$-F_c) contoured at 1.5 σ are shown in blue.

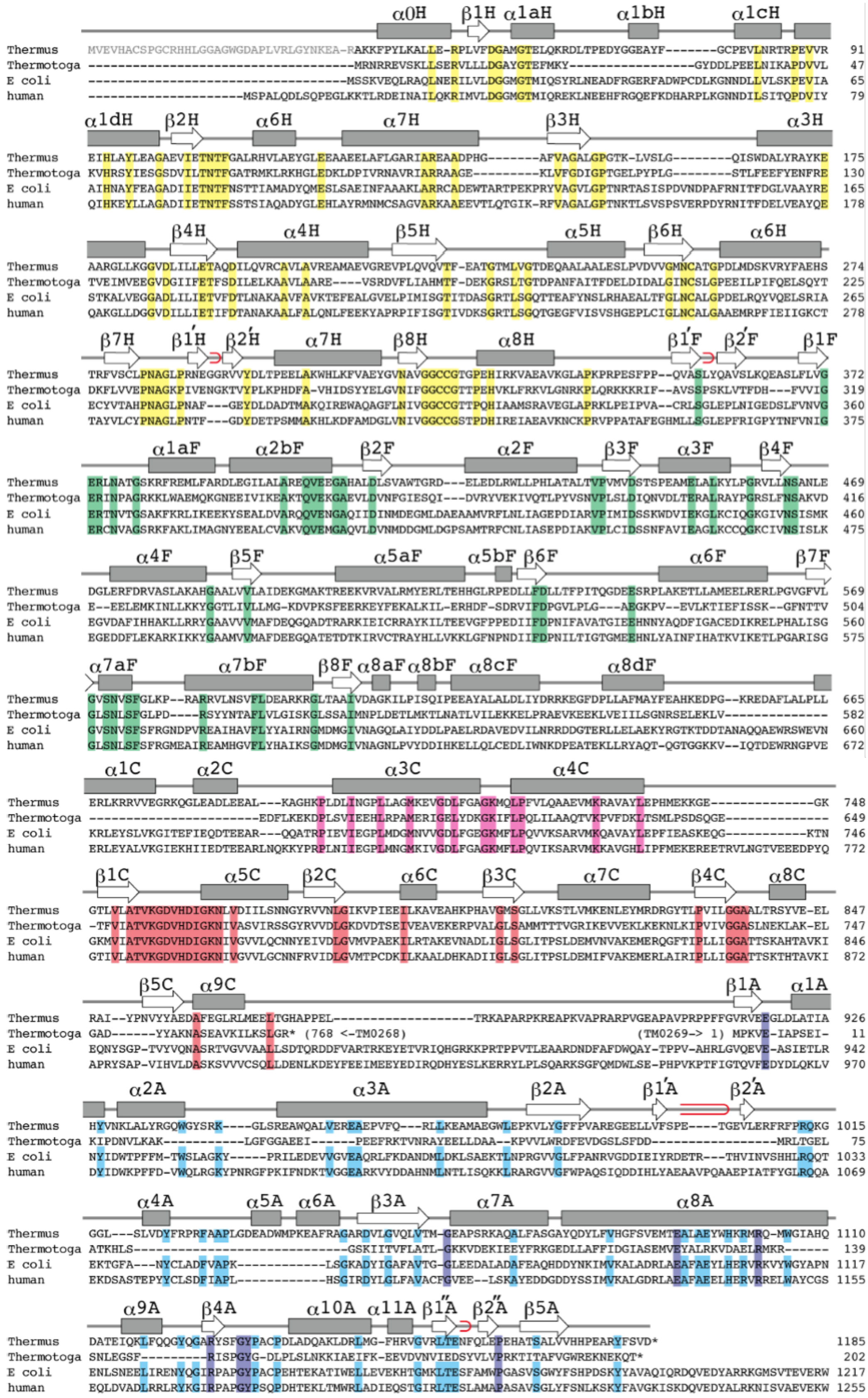
Thermus
Thermotoga
E coli
human
α0H β1H α1aH α1bH α1cH
α1dH β2H α6H α7H β3H α3H
β4H α4H β5H α5H β6H α6H
β7H β1′H β2′H α7H β8H α8H β1′F β2′F β1F
α1aF α2bF β2F α2F β3F α3F β4F
α4F β5F α5aF α5bF β6F α6F β7F
α7aF α7bF β8F α8aF α8bF α8cF α8dF
α1C α2C α3C α4C
β1C α5C β2C α6C β3C α7C β4C α8C
β5C α9C β1A α1A
α2A α3A β2A β1′A β2′A
α4A α5A α6A β3A α7A α8A
α9A β4A α10A α11A β1″A β2″A β5A

Supplementary Figure 8. Methionine synthase (MS) sequence alignment. Schematic illustration of the secondary structure of *t*MS and alignments with the amino acid sequence of MS from *Thermus thermophilus* [Genbank accession number NC_00646], *Thermotoga maritima* [NC_000853], *Escherichia coli* [J04975], and human [U73338]. α-helices and β-sheets are shown in boxes and arrows, respectively. Red loops indicate β-hairpins. The N-terminal residues absent in *t*MS$^{\Delta N35}$ are shown in gray. Conserved amino acid residues are highlighted by yellow, green, pink, red, and blue for the homocysteine, folate, Cap, cobalamin, and *S*-adenosylmethionine domains, respectively. In the activation (Act) domain, cyan is used to highlight conserved amino acid residues in *T. thermophilus*, *E. coli*, and human MS.

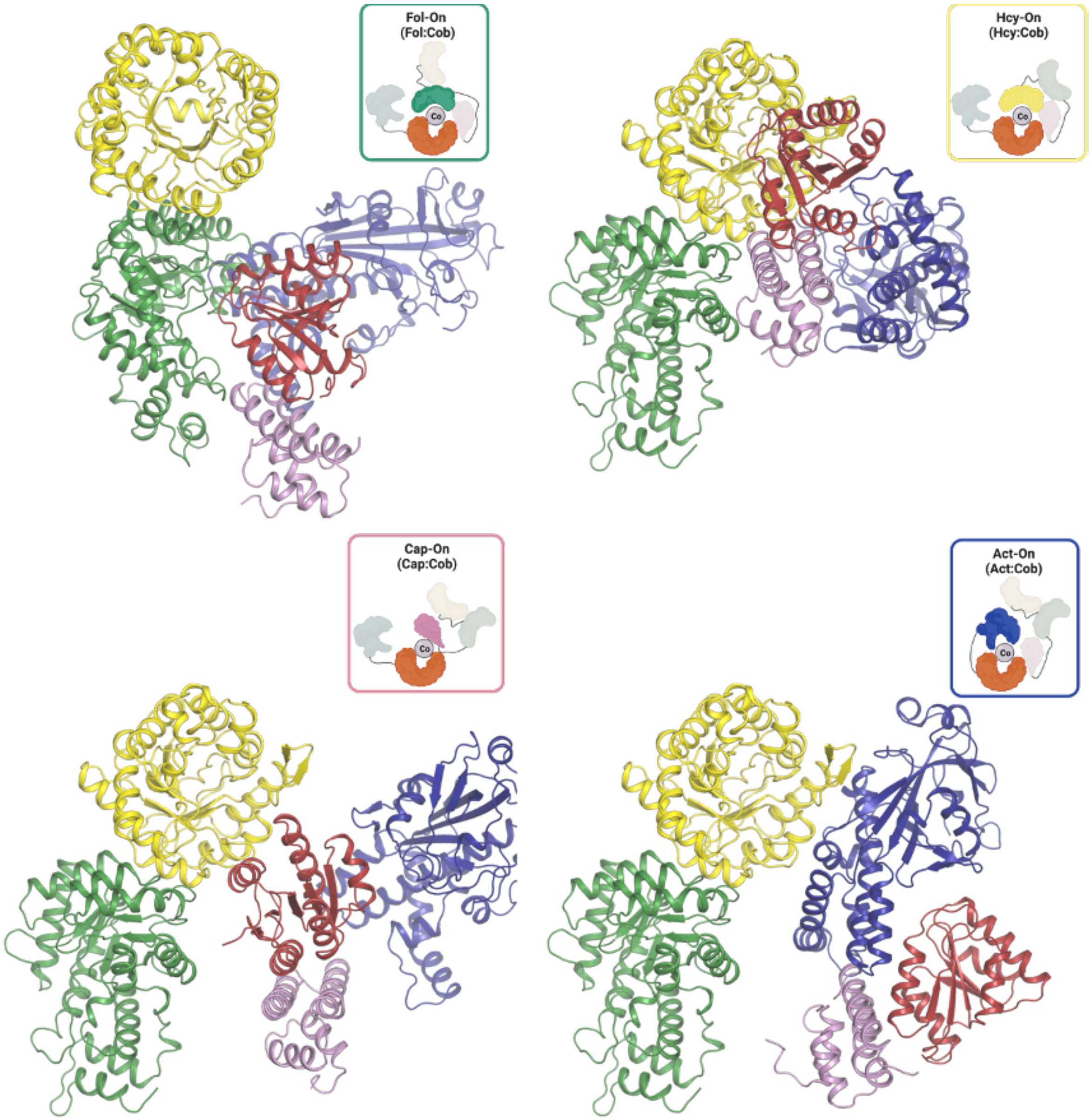

Supplementary Figure 9. Conformational dynamics of MS. The proposed conformations adopted by MS throughout the catalytic and reactivation cycles with different substrate binding domains positioned above the cofactor; Fol-on (top-left), Hcy-on (top-right), Cap-on (bottom-left), and Act-on (bottom-right). Figure insets created with BioRender.com.

Supplementary Tables

Supplementary Table 1. Kinetic parameters of Methionine Synthase

Enzyme	Molecular Weight (kDa)	Specific Activity (μmol min^{-1} mg^{-1} protein)	k_{cat} (min^{-1})	K_m (μM)	
				(6S)CH_3-H_4folate	Homocysteine
E. coli MS	133	11.6[1]	1542 at 37°C<br>1128 at 25°C[2]	27.8	0.8
T. thermophilus MS	131	8.1	1062 at 50°C	18.4 ± 4.1	9.3 ± 2.8
S. scrofa MS (Pig)	150	1.7	255	12.6	2.16
R. rattus MS (Rat)	140	1.6	224	38	1.7

[1]Biochemistry 1998, 27, 8458-65
[2]Biochemistry 1990, 29, 11101-9

Supplementary Table 2. X-Ray Data Collection and Refinement Statistics

	*t*MS$^{\Delta N35}$	*Apo-t*MS$^{Cap:Cob:Act}$	*Holo-t*MS$^{Cap:Cob:Act}$
**Data collection**			
Beamline	APS, GMCA 23-IDB	APS, GMCA 23-IDB	APS, GMCA 23-IDB
Wavelength (Å)	1.033	1.033	1.033
Temperature (K)	100	100	100
Resolution (Å)	106.64-2.75 (2.85-2.75)*	40.55-2.40 (2.50-2.40)*	49.30-3.15 (3.23-3.15)*
Space group	$P4_12_12$	$P3_121$	*C*121
Cell dimensions			
a, *b*, *c* (Å)	134.84, 134.84, 174.74	96.18, 96.28, 356.04	166.14, 95.84, 238.75
α, β, γ (°)	90, 90, 90	90, 120, 90	90, 91.96, 90
Observed reflections	669,916 (51,333)	711,608 (42,258)	222,439 (11,567)
Unique reflections	42,467 (4,373)	76,051 (4,396)	64,264 (3,294)
R_{meas} (%)	19.7 (167.3)	18.7 (95.4)	22.0 (116.7)
R_{merge} (%)	19.1 (160.0)	17.7 (90.3)	18.5 (98.6)
<I/σ>	12.3 (1.8)	8.1 (2.5)	5.1 (1.3)
CC(1/2)	0.993 (0.672)	0.99 (0.667)	0.99 (0.581)
Multiplicity	15.8 (11.7)	9.4 (9.6)	3.5 (3.5)
Completeness (%)	100.00 (100.00)	100.00 (100.00)	98.50 (99.22)
Wilson *B*-factor (Å^2)	56.2	47.00	60.70
**Refinement**			
Resolution (Å)	106.96 - 2.75	39.85 - 2.95	49.30 - 3.15
No. reflections	40,263 (2,154)‡	75,954 (3,830)‡	64,264 (3,294)‡
R_{work}/R_{free} (%)	22.6/25.3	26.5/29.9	24.6/30.1
No. of non-H atoms			
Protein	8751	12,223	24,885
Water	131	493	299
Ligand	11	0	546
B-factors (Å^2)			
Protein	81.63	40.63	84.67
Water	58.80	44.00	44.50
Ligand	133.13	0.00	70.93
R.m.s. deviations			
Bond lengths (Å)	0.008	0.0075	0.012
Bond angles (°)	1.36	1.70	1.99
Ramachandran Plot			
Favored/allowed/outliers	96.9/3.0/0.1	97.5/2.5/0.0	97.9/2.1/0.0
MolProbity Score	1.03 (100th percentile)	0.60 (100th percentile)	0.99 (100th percentile)
PDB	8SSC	8SSD	8SSE

* Highest-resolution shell is shown in parentheses.

‡ Number of reflections used for cross-validation

Supplementary Table 3. Purification Yield and Activity

	**Protein*** **(mg)**	**Total activity (μmol min^{-1})**	**Specific Activity (μmol min^{-1} mg^{-1} protein)**	**Purification *n*-Fold**	**Yield (%)**
Crude Extract	674#	700	1.04	1.0	100
Heat treatment	218	390	1.80	1.7	56
Ni-affinity Chromatography	37	300	8.11	8.0	44

*Protein concentration was determined using the Bradford method {Bradford, **1976**, 2300}.
CH_3-cobalamin was added when activity was measured.

Supplementary Table 4. **Bacterial strains, plasmids, and primers used in this study.**

Strains, plasmids, and primers	Relevant characteristics	Ref. or sources
***E. coli* strains**		
XL1-Blue	routine cloning strain, tetracycline resistance	Stratagene
BL21star(DE3)	widely used T7 expression strain, no antibiotic resistance	Invitrogen
Plasmids		
pET11a-*t*MS^{wt}	wild-type *t*MS in pETlla vector, containing the *T. thermophilus* gene *MetH*, resistant to ampicillin	Riken
pMCSG7-*t*MS^{wt}	*t*MS in pMCSG7 *E. coli* expression vector encoding an N-terminal His-tag with TEV cleavage site, resistant to ampicillin	This Work
pMCSG7-*t*MS$^{\Delta N35}$	*t*MS N-terminal 35aa truncation (ΔN35)	This Work
pMCSG7-*t*MS^{CobAct}	*tMS* C-terminal half, containing Cap, Cob, and Act domains (CobAct)	This Work
Primer Names	Sequences	Descriptions
*t*MS_f	5'- ACTTCCAATCCAATGCCATGGTGGAGGTCCA-CGCCTG - 3'	LIC, wt, vector
*t*MS_ΔN35_f	5' - TACTTCCAATCCAATGCGAAGAAGTTTCCCT-ACCTCAAG - 3'	LIC, ΔN35
*t*MS_L660+LIC_f	5' - TACTTCCAATCCAATGCCCTGGCCCTTCCCC-TCCTGGAG - 3'	LIC, CobAct
*t*MS_LIC_r	5' - TTATCCACTTCCAATGCTAGTCCACGCTGAA-GTAGCG - 3'	LIC, vector
